# Lunacy *versus* Sanity—Law *versus* Physic

**Published:** 1830-04-01

**Authors:** 


### Periscope;

OR,

### CIRCUMSPECTIVE REVIEW.

" Ore trahit quodcunque potest, atque addit acervo."

### I.

### Lunacy *versus* Sanity—Law *versus* Physic.

" Maxima inter has differentia; et tot doctorum sententiæ, quot ipsi numero sunt." *Avicenna.*

In the late remarkable trial of Mr. Davies for the crime of insanity, LAW has not gained; but PHYSIC has decidedly lost ground! And why should we wonder that the bar, the bench, the college, the jury, should all wrangle, and dispute, and disagree about INSANITY, when they are all mad themselves! Every body knows that CHARON, according to the testimony of Lucian,* was conducted by MERCURY to a very high hill, (Mont Blanc, or Chimborazo, perhaps) from whence he had a view of all the nations of the earth. Charon eyed the immense prospect very attentively, and then Mercury, that witty chap, (Nepos Atlantis) inquired of him what he saw? The answer was, that he could discover cities like bee-hives, each bee having a *sting*—that the bee-men did nothing but *sting* one another—that over their heads hovered a tumultuous multitude of passions in the shapes of hope, fear, anger, avarice, envy, &c. together with innumerable diseases which they were always pulling down on their own heads! Some of the bee-men appeared to him to be brawling, some fighting, some running, some riding, ("solicite ambientes, *calide litigantes*") and contending for all manner of toys and trifles. In conclusion, he pronounced them all to be *madmen*, fools, and asses! " Insana studia (he exclaims) insani labores—mad endeavours! mad actions! mad! mad! mad!"

All our medical readers know that when Hippocrates was sent to see Democritus (as Dr. M'Michael was sent to see Mr. Davies) he received such sapient answers from the supposed lunatic (whom by the bye, he found dissecting animals to discover the *cause of madness*) that he declared to the citizens of Abdera that, notwithstanding some *small eccentricities*, " the world had not a wiser or more honest man than Democritus." So Dr. M'Michael wisely concluded that Mr. Davies' lessons in boxing were proofs of sanity, being very natural and proper precautions against the pugilistic propensities of the tea-dealers in Philpot-lane.

Why should we attach any weight to the testimony of a jury respecting the madness of others, when it is asserted, upon the very best ancient authority, that *we are all mad?*

" Et semel, et simul, et semper insanivimus omnes."

SOCRATES searched every where and could not find a wise man—and when we consider that every deviation from wisdom, judgment, sense, must be a grade, however small, of insanity,—when we reflect that the whole of the passions and propensities of our nature have a tendency, a constant tendency, to derange the mind—and not only the passions within us, but almost all the external circumstances by which we are surrounded, we shall, on an accurate survey of human nature, and serious reflection on its state and condition in this world, come to the same conclusion as the ancient sages, that there

* Dial. Contemplantes.

is no man in his proper senses. It may not be useless or unentertaining, though a melancholy subject, to glance at some of the principal causes which produce, according to the degree of their intensity, an aberration, more or less marked, from a sane state of the intellect.

The IMAGINATION is constantly leading us into shades and grades of madness. How many chimeras are engendered by the force of imagination! How many golden day-dreams float along the sensorium, even in the murky atmosphere of Cornhill! How many golden mountains have been raised among the Andes, that had no other existence than in the imagination? Can those men be pronounced sane who have squandered away their fortunes and ruined their families on chimerical speculations — or those who were weak enough to entrust their property in the hands of madmen? What act of Mr. Davies' life was half so insane as hundreds of acts lately performed, not merely by individuals, but by whole companies of collected wisdom?

SORROW and GRIEF are designated the mother and daughter of melancholy. Hippocrates tells us that sorrow begets insanity, and insanity sorrow—that they tread, as it were, in a circle of production and reproduction. Solomon says that sorrow "dries up the bones"!

Tanta illi est feritas, tanta est insania luctus.

*Fear, envy, anger, malice, hatred, emulation, jealousy, revenge, ambition, discontents, cares, misfortunes,* and ten thousand other causes are perpetually operating to drive SOUND REASON from its just and proper path, and thus to effect more or less aberration of the intellect. Every ECCENTRICITY, as it is called, and as the term literally imports, is a divergence from sanity of mind. When these eccentricities are slight—harmless — droll, they only produce amusement to the rest of the community, and are not considered as insanity. But whenever any of the various causes above-mentioned happen to operate more powerfully than usual—or when bodily disorder comes to aid original obliquity of mind, then the eccentricity becomes more prominent; and when this aberration happens to interfere with the interests of others, a commission of lawyers and doctors is forthwith summoned, to try on which side of the line of demarcation between eccentricity and insanity, the individual is placed. Is it very wonderful that a junto of men, each having his own personal hobby, humour, eccentricity, *idola specus*—or, in other words, MONOMANIA, should be all at sea in determining the above-mentioned boundary, when the subject of the investigation is just hovering between the two ill-defined, and indeed indefinable regions?

The late memorable commission of lunacy, in Mr. Davies' case, collected together, in Grays Inn Lane, the greatest number of madmen which we ever remember to have seen in any one place, if we except certain congregations at the FREE MASONS' TAVERN, a few years ago, when Mr. Wakley aspired to the elevated character of legislator, before he fell back into the humble sphere of church-warden in the parish of St. Giles. That the party who *prayed* for the commission, were as mad as the frogs who *prayed* to Jupiter for a king, might be proved by at least 5000 good reasons, which, ere now, are engrossed by the attornies in very legible, though perhaps not very intelligible characters! As for the lawyers on that side, they were decidedly *monomaniacal!* Excepting indeed on the single point of fees for themselves, they appeared to be in a state of general insanity. Who but madmen would have produced a "TRASH-SPOUTER," as Mr. Adolphus aptly termed him—a common hack-witness, who travels from county to county, to dole out the jargon of Bedlam, within whose walls he was so long an inmate, for the purpose of forcing an inoffensive chewer of Souchong or Wang-quang tea, in Philpot Lane, to take up his abode in the "RETREAT" on Clapham-rise rather than on Crouch-hill! Who but madmen would have called before the commissioners, a physician who swore that it was *madness* in Mr. Davies to "lock up" five or seven thousand pounds in a snug estate, although he swore, at the same time, that he knew not what was the amount of property possessed by the buyer of that estate! To our minds, and no doubt to those of the jury, the act of "locking up" five or seven thousand pounds, was a much stronger proof of sanity than the act of taking lessons in boxing, which Dr. M'Michael considered unequivocal evidence of sound mind. If Sir Charles Wetherell looked on this "*locking up*" propensity as *disqualifying* poor Davies for the management of his own affairs, why did he call as evidence, Dr. Burrows and Sir George Tuthill, who are themselves notorious for this same "*locking-up*" propensity; and who were, according to his own shewing, *disqualified* witnesses?

The lawyers on Mr. Davies' side, though they shewed themselves much less insane than their opponents, are not quite free from suspicion of intellectual obliquity. Many of Mr. Brougham's observations on the case were far from being wise, however witty they might appear. Who but a MADMAN would object to the testimony of "MAD DOCTORS," in cases of insanity? Is it not an established maxim, in jurisprudence, as well as in every other kind of prudence, to—"set a thief to catch a thief."* Why then should Mr. Brougham have borne so hard on the "mad doctors"—and especially on the "*spouter of trash,*" whom he pro-

* The Editor of this Journal was very much amused, one day, while visiting the Lunatic Asylum, in Florence, (where it is the custom to send all insane people, whatever their station in life) by the conversations of a Priest and an Advocate, who were monomaniacs of such a harmless description that the keeper permitted them to accompany the Editor round the whole of the wards and cells of that great and wretched asylum. These two inmates of this gloomy retreat were men of considerable talents and learning. They described, in most affecting terms, the various maniacs who paced the wards in musing melancholy or muttering soliloquies, as well as those who clashed their chains in solitary confinement. Not a word escaped either of them, in the slightest degree indicative of a disordered mind, till they came to a man, who fancied himself to be JESUS CHRIST. The Barrister made a full stop, and seized the writer by the arm. "Thank my stars," said he, glancing a look of ineffable contempt on the Priest, "I am free from those superstitious fears and visionary dreams by which the vulgar are kept in thraldom by designing knaves or ignorant enthusiasts! I worship the sun, the moon, the stars, the earth—in short, I worship NATURE, whatever form she may assume in the animal, vegetable, and mineral world around me, as well as in those orbs which shine resplendent in the heavens. I acknowledge no god but Nature."—At this moment, the Priest seized the other arm of the writer, and drew him forcibly aside. "You now see, Sir," said he, "that the unhappy and lost wretch who deals out this impious and atheistical creed is as complete a maniac as any of the numerous unfortunate beings whom we have been contemplating! He is otherwise harmless; but his words are pestilential, when he touches on the subject of divine revelation. I am, Sir, the only individual in this vast asylum, who is in his perfect senses. I am cruelly and unjustly confined here, and, as I see you are a physician, I hope you will exert your influence in rescuing me from the company of maniacs." The writer promised this exertion in the Priest's favour, but soon found that he, too, had his delusion. It turned out that the Barrister was not so insane upon religious topics as on some others. His insanity was first discovered by a pathetic love-letter to a beautiful young English lady at Florence! It is notorious that the insane are perfectly conscious of the madness of their comrades, while they are totally blind to their own. But is not this the case with every eccentricity—every obliquity of intellect—every failing of human nature?

nounced to be past redemption? Now, although this "*trash-spouting*" propensity may be Dr. Haslam's hobby, eccentricity, monomania, or whatever other species of intellectual obliquity we may chuse to call it; still it constitutes no small item of his support, whether it be uttered, *viva voce*, while walking the circuits, or weekly vomited forth, as is pretty strongly suspected, from the Lancet Office. Mr. Brougham has done Dr. Haslam a serious injury in thus lopping off, for ever, his office of "walking witness" to the commissions of lunacy.

We shall say but a few words respecting the jury. We would not swear that they were "compos mentis," in deciding that a man was perfectly sane, after fourteen or fifteen medical men (most of them mad doctors, who can scent insanity as unerringly as a vulture can scent carrion) swore that he was insane! True, they had the evidence of two doctors in favour of Mr. Davies' soundness of mind; but these evidences were by no means strong—they were negative rather than positive, and we beg to suggest that one unequivocal instance of insanity being proved, is worth twenty interviews where the hallucination is not forth-coming. We sincerely hope that time may prove the jury to be sane.

Of Dr. M'Michael, who played his part so very cleverly, we entertain some suspicions; and we are not quite sure that even he did not labour under a "temporary delusion." The Doctor stated on oath, that he received the deposition of Dr. Burrows, with the Lord Chancellor's order to visit Mr. Davies. That he read this report, cannot be doubted, because he refers to two of the points of delusion under which Mr. D. was there stated to labour. Many other points of delusion were recorded in Dr. Burrows' report; yet Dr. M'Michael, in his cross examination, declares that he heard nothing of *these other points of hallucination* till they came out in court. Dr. M. is a subtle casuist, and no doubt he will be able to clear up this knotty point.

But of all the mad doctors assembled on this mad investigation, Dr. M'Kinnon appears to have been the most mad—or the most modest, which is the same thing in physic. What a glorious opportunity has he lost of obtaining a legitimate puff of the very first water! When asked — when even *pressed* to state the amount of his fees, or in other words, the extent of his practice, he stood more coy and modest than Goldsmith's village maid, for she was "*half-willing to be pressed*"—and thus he neglected to turn to account one of those golden chances, for which *some* of his confreres (with half his practice) would have given a year's fees! O fie Dr. M'Kinnon, you will never succeed in modern Babylon!

Of all the Dramatis Personæ, in this memorable transaction, there is only one whom we can pronounce to be sane—and that because a *jury*, rather than judges, determined him to be so. That Mr. Davies *had been* on the wrong side of the line which is supposed to separate eccentricity from insanity, we believe no man on the right side of that line will deny—that he was perfectly recovered when the ordeal took place, we have doubts — and that he has strength of mind to bear this splendid victory over his opponents, we have fears. We have lived long enough in this world to know, that evil, to a strong mind, very generally proves in the end a benefit — while, to weak minds success is the greatest curse.

Admiring, as we do, the laws of our native land, yet, considering that they were framed by men, we suspect they are not perfection. Insanity is a disease requiring the light of medical science to ascertain its existence, when arrayed in dubious colours. The law says that the only ligitimate judges of the disease shall be exempted from serving on juries. Does this infer that no 12 honest medical men could be found to hear the evidence respecting sanity or insanity, and afterwards to examine the individual, whose mental powers were subjected to scrutiny? A man may be immured in a lunatic asylum on the faith of a single medical practitioner—and a medical practitioner *only*;—but when a solemn investigation takes place, as to the true state of the man's mind, the jury (who are erected into judges) are composed of men having no knowledge whatever of the nature of the subject they are to decide upon ! ! If it be said that the jury have the advantage of medical witnesses, let the late trial be contemplated. The jury decided *against* 15 medical witnesses, before they heard a single witness on the other side of the question ! We think there can be little doubt that this hasty, we had almost said *ex parte*, decision arose from the insanity of some of the medical witnesses, who, in the minds of the jury, were fitter subjects for seclusion on Clapham Rise than Mr. Davies. We apprehend that this trial will prove an æra in the transactions of lunacy commissions. Lawyers are tolerably acute. The supporters of a commission "de lunatico inquirendo," will be somewhat cautious how they pack a witness box with mad doctors and ragged itinerant evidence ! The human mind revolts against a display of strength collected apparently for the purpose of gaining a legal victory, rather than for the establishment of truth, the interests of society, and the protection of personal liberty.

